# A dismantling study on imaginal retraining in smokers

**DOI:** 10.1038/s41398-020-01191-9

**Published:** 2021-02-02

**Authors:** Steffen Moritz, Josefine Gehlenborg, Janina Wirtz, Leonie Ascone, Simone Kühn

**Affiliations:** 1 Department of Psychiatry and Psychotherapy, University Medical Center Hamburg-Eppendorf (UKE), Hamburg, Germany; 2 Lise Meitner Group for Environmental Neuroscience, Max Planck Institute for Human Development, Berlin, Germany

**Keywords:** Addiction, Neuroscience

## Abstract

Imaginal retraining is a noncomputerized variant of cognitive bias modification, an intervention aimed at reducing craving in substance use disorders and behavioral addictions. We conducted a dismantling study to elucidate which of its multiple components are effective and hence essential ingredients of the training and which are ineffective (and hence perhaps dispensable) in reducing craving. We randomized 187 smokers to one out of six conditions that instructed participants to perform a brief intervention. In four of these, participants were instructed to perform isolated components of the imaginal retraining protocol, and in the two other conditions participants either suppressed or simply observed (control condition) the image of a cigarette. Before and after the intervention, participants were asked to rate their level of craving and how pleasant they found three smoking-related images. We examined within-group changes by means of paired *t*-tests separately across conditions (trial registration: DRKS00021044). Mental distancing from cigarettes (without a corresponding actual physical movement; non-motor retraining) led to a significant decline in craving (paired *t*-test), which remained significant when compared to the control condition. The effects of other components of the retraining were less consistent. The present study shows that a single therapeutic “dose” of distinct components involved in imaginal retraining can reduce craving for cigarettes. Future trials should investigate the effectiveness of components of imaginal retraining not yet tested (e.g., mood induction) and whether combinations and repetition of single components strengthen or dilute efficacy.

## Introduction

### Smoking—a smoldering challenge

Smoking is a prevalent substance use disorder (SUD), especially in Europe^1^, and it is accompanied by severe and life-shortening health consequences^2,3^. Smoking causes a loss of ~150 million disability-adjusted life years (DALYs)^4^ and is one of the five leading risk factors for loss of DALYs worldwide, which has sparked calls for enhanced political commitment to prohibiting smoking and to searching for new treatment methods^5^. According to a large epidemiological study in Germany with 12,273 participants^6^, almost all smokers have made at least one attempt to quit in the last 12 months, with only 12.5% of these applying evidence-based methods. Most smokers try to quit unassisted^6^, which rarely leads to sustainable abstinence^7,8^. Failure to quit has been attributed to craving and withdrawal symptoms^9^. Consequently, there is a need to find new treatments and raise the efficacy of low-threshold (self-help) treatment options.

### Automatic and controlled processing in substance use disorders

According to the dual-process model, behaviors are determined by both automatic and controlled processes^10^. Whereas anxiety disorders are characterized by pathological automatic avoidance of certain (often harmless) situations or objects, addictive disorders have been explained by an exaggerated automatic approach bias for unhealthy substance-related objects such as cigarettes, mostly in the absence of conscious control^11^. Smokers show attentional biases toward smoking-related cues; they notice such cues more quickly and pay more attention to them than non-smokers do^12,13^. The present study is concerned with imaginal retraining, a self-help variant of cognitive bias modification (CBM). CBM and imaginal retraining aim to reduce craving in SUDs as well as behavioral addictions by reducing the underlying approach bias^14^. Smokers are quicker to approach than to avoid smoking-related stimuli and demonstrate an approach bias toward smoking-related cues^13,15–18^. Studies suggest a correlation between a craving for tobacco and the approach bias in smokers^19,20^. Moreover, it has been demonstrated that CBM could reduce the approach bias for smoking-related cues and might also facilitate smoking cessation^15^. Whereas CBM is carried out via a computer (whenever an image of an addictive substance is displayed, the user pushes a joystick that decreases the size of the picture; in contrast, in response to positive or neutral images, the user pulls the joystick, which enlarges the image and creates the impression of approach), imaginal retraining is a noncomputerized technique that merges imaginal and behavioral elements. The participants are first asked to imagine a substance they crave (e.g., cigarettes), which is followed by a negative mood induction. In the mind’s eye, the user then throws the craved object away (note that this sequence corresponds to the push movement in CBM). This is accompanied by a forceful in vivo throwing movement (see the online Appendix for a visualization). This sequence alternates with a counter-movement in which the user first imagines a tasty but non-addictive beverage or food coupled with other positive images and feelings. The food or beverage is then brought toward the user and is consumed in a somewhat exaggerated way to increase the feeling of relish. The effectiveness of imaginal retraining has been demonstrated in smokers^21^, people with alcohol use disorder^22^, and overweight individuals who crave high-calorie food^23^. A possible disadvantage of the original CBM technique is that the procedure can be boring for some individuals^24^ and requires certain technical devices, which makes routine implementation more difficult. Moreover, the set of stimuli used in these computerized procedures is limited and difficult to personalize, particularly with regard to the typical environment of intake of the addictive substance.

### Possible mechanisms of cognitive bias modification and imaginal retraining

Central to both CBM and imaginal retraining is the concept of embodiment, which assumes a strong interrelation of bodily motions, emotions, and cognitions^25,26^; in line with this, the perception of a stimulus and its mental simulation map onto similar sensorimotor cortical areas^27–29^. Embodiment is actively conveyed to participants in imaginal retraining as a mode of action, which is intended to raise self-efficacy and thus ignite more explicit psychological processes.

Like the original technique, imaginal retraining consists of multiple components: a push/pull movement, confrontation with a desired (addictive) versus non-addictive substance/object, and zooming in and out (the objects grow larger or smaller in size when pulling or pushing). To date, it is unclear which of these components contribute most strongly to the anti-craving effects of CBM and imaginal retraining. Moreover, it is unclear whether the technique works only after repeated rehearsals (i.e., automatization of avoidance movements) or whether a single “dose” (i.e., a single exercise) can reduce acute craving. The latter question is important as many individuals with substance abuse and behavioral addictions have problems with immediate craving and may not want to or are unable to engage in a lengthy training procedure before experiencing a reduction in craving.

Alongside anti-craving medication, examples of coping strategies for acute craving are thought suppression and acting with awareness (AWA). Whereas suppression aims at turning one’s attention (almost compulsively) away from the craved stimulus, AWA consists of mindfully turning toward the stimuli with kindness and curiosity^30^. Research has shown that although suppression can reduce cravings in the short term^31^, it is also likely to lead to rebound effects and heightened craving in the long run^32,33^. Mindfulness-based relapse preventions, on the other hand, aim at coping with cravings by acting with awareness^34^ and have been found to be an effective coping mechanism in SUD^35^.

### The present study

Within the framework of a dismantling study, we examined the effect of single components of imaginal retraining relative to a neutral condition (just observing an image of a cigarette) as well as a thought suppression condition. Immediately before and after the intervention, we asked smokers to rate their level of craving, which served as the primary outcome, and also their appraisal of smoking-related pictures. We expected that the core retraining components equivalent to the push movement of the joystick in CBM would be associated with the largest reductions in craving.

## Methods

### Sample

The present study was conducted as part of a 1-year follow-up assessment of smokers who had participated in a randomized controlled trial comparing imaginal retraining and two control conditions (*N* = 345; wait-list and active control; the study was sufficiently powered to detect a small effect size). Prior group allocation was considered a moderator.

Initial inclusion criteria were age between 18 and 75 years and current self-reported smoking. A formal diagnosis of tobacco dependence or fulfillment of any threshold criteria was not mandatory. For further details on recruitment, mode of assessment, and the initial technique, see the original study^21^.

The initial and the follow-up trials were registered with the German Clinical Trials Register (DRKS00016860, DRKS00021044). Ethical approval was obtained from the local psychological ethics committee of psychologists of the University Medical Center Hamburg (Germany; LPEK-0023; LPEK-0104).

### Invitation and survey

All participants from the baseline assessment were re-invited; a total of 187 participants took part in the dismantling experiment of the follow-up assessment. Importantly, the present experimental conditions were not described in detail in the invitation letter so that lack of interest or belief in the efficacy of acute anti-craving methods could be ruled out as reasons for (non-)participation. Participants were told only that they would be shown pictures of cigarettes that they would rate and respond to using certain strategies.

As in the baseline and post surveys, assessments were carried out online using Questback/UniPark^®^. In accordance with the guidelines of the European General Data Protection Regulation, no IP addresses were stored. After reading the welcome page, participants gave their electronic informed consent. Afterward, they were asked to provide an anonymous email address for receiving the invitation to participate. Subsequently, smoking questionnaires and other psychological scales were administered (see below). After completion of the follow-up measures, each participant was assigned randomly to one of the six experimental conditions (see the “Experiment” section). Last, the participants had to indicate whether they had answered all questions truthfully.

### Questionnaires

Only the questionnaires that are directly relevant to the study are described here.

The Cigarette Dependence Scale (CDS-12, CDS-5)^36^ is a self-rating questionnaire measuring nicotine dependency according to DSM-IV and ICD-10 criteria. We evaluated both the long (12 items) and the short (5 items) versions of the CDS. The validity and reliability of the scale are good^37,38^.

The Fagerström Test for Nicotine Dependency (FTND)^39^ is a self-rating questionnaire composed of six questions measuring the severity of addiction, with scores ranging from 0 (= low dependence) to 10 (= very strong dependency). The FNTD has been shown to be reliable^40^.

### Experiment

In the last section of the survey, participants rated their current level of craving for cigarettes on a scale ranging from 0% (not at all) to 100% (extreme). This was followed by three smoking-related pictures (a hand holding a lit cigarette; an open, full box of cigarettes; and two people smoking outside in a relaxed and pleasant atmosphere; see Appendix), which participants rated from 0 (= repelling) to 100 (= very pleasant) via a slider (10 point steps). Participants were then randomized to one of six conditions (see the Experimental conditions section); the allocation was unknown to the investigators. The intervention was followed by the administration of the Patient Health Questionnaire-9 (PHQ-9)^41^ as a filler scale (no results were calculated). Subsequently, participants were asked whether they had actually executed the designated task. Participants were assured that a “no” response would have no negative consequences. Then, participants rerated the same three pictures in the same order as before. Afterward, they were asked whether they had either experienced no change in craving or a real change in craving or had endorsed a change in ratings because they felt this was expected of them or for a different reason (with free-text format so they could specify the reason). Subsequently, participants indicated their current level of craving for cigarettes. At the end, we asked whether they had truthfully answered the questions (yes/no), and completers were rewarded with a manual containing relaxation and self-compassion exercises.

### Experimental conditions

Six conditions were administered in random order (determined by computer algorithm). Interventions 1–5 consisted of short instructions on what participants should do while seeing a picture of a lit cigarette (see the Appendix); for intervention 6, participants viewed an image of a woman drinking a bottle of water.

1. Control condition: “Please take a quick look at the cigarette. Only press “continue” after you have actually followed this instruction.” (word count of original German text: 18)
2. Thought suppression: “Please do the following: Look at the picture, then close your eyes and try not to think about the picture for a few seconds. Please carry out this exercise even if it feels a bit awkward. Only press “continue” after you have actually followed the instructions.” (word count of original German text: 56)
3. Zooming out: “Please do the following: Look at the picture. Then close your eyes and imagine the picture slowly getting smaller. Please carry out this exercise even if it feels a bit awkward. Only press “continue” after you have actually followed this instruction.” (word count of original German text: 55)
4. Imagining to throw + zooming out + movement (imaginal retraining with motor component): “Please do the following: Look at the picture and imagine yourself throwing the object in the picture away from you in your imagination and the picture slowly becoming smaller. Please carry out the actual arm movement as if you were throwing away the cigarette or a whole pack of cigarettes with contempt. Please carry out this exercise even if this feels a bit awkward. Only press “continue” after you have actually followed the instructions.” (word count of original German text: 78)
5. Imagining to throw + zooming out (imaginal retraining with no motor component): “Please do the following: Look at the picture and imagine yourself throwing the object in the picture away from you in your imagination and the picture slowly becoming smaller. Execute the movement in your imagination only. Please carry out this exercise even if it feels a bit awkward. Then press “Next.” (word count of original German text: 59)
6. Counter-movement (see picture in the Appendix): “Please do the following: Imagine that you are having a delicious drink and that you are holding the drink a bit higher than usual—like in the picture—so that your gaze is directed slightly upward. Please carry out this exercise even if it feels a bit awkward. Only press “continue: after you have actually followed the instructions.” (word count of original German text: 64).

### Statistical analysis

We planned to conduct pre-post comparisons to elucidate significant effects on participants’ craving and their appraisal of smoking-related pictures. Subsidiary analyses also examined the effects of single pictures. We also planned direct comparisons between the retraining conditions (#4 and #5) with the control condition (#1). Due to the exploratory nature of the trial, we did not correct for multiple comparisons (see the “Discussion” section). Data are available upon request.

## Results

Sample characteristics are displayed in Table 1. Participants were mainly in their late 40s/early 50s and showed rather low dependency on the FTND. No group differences occurred regarding any of the background characteristics (see Table 1).

**Table 1:** Demographic and smoking-related characteristics of the sample assigned to the six conditions (*N* = 187).

	Control (*n* = 33)	Thought suppression (*n* = 35)	Zooming (*n* = 28)	Retraining with movement (*n* = 30)	Retraining without movement (*n* = 33)	Counter-movement (*n* = 28)	Statistics
Age in years	49.15 (11.38)	52.00 (11.74)	52.93 (9.57)	48.33 (13.06)	50.27 (11.55)	52.54 (12.88)	*F*(5, 181) = 0.79, *p* = 0.561
Gender (male/female)	14/19	17/18	15/13	19/11	12/21	16/12	*χ*^2^(5) = 8.65*, p* = 0.124
Number of daily cigarettes	11.67 (10.56)	10.60 (10.17)	9.68 (10.94)	7.43 (8.40)	11.42 (12.23)	9.18 (8.36)	*F*(5, 181) = 0.74, *p* = 0.594
FTND	3.21 (2.90)	2.80 (2.56)	2.54 (2.90)	2.17 (2.60)	2.61 (2.56)	1.82 (2.16)	*F*(5, 181) = 1.04, *p* = 0.395
CDS-12	36.42 (13.87)	32.20 (12.93)	32.04 (15.10)	29.80 (13.91)	36.52 (13.56)	33.04 (13.66)	*F*(5, 181) = 1.17, *p* = 0.325
CDS-5	15.06 (5.89)	13.89 (5.09)	13.54 (6.07)	12.40 (5.18)	14.94 (5.21)	13.64 (5.01)	*F*(5, 181) = 1.04, *p* = 0.395

Frequency (gender), means and standard deviations (in brackets).

*BDI* Beck Depression Inventory, *CDS-12* Cigarette Dependence Scale (*12 items), CDS-5* Cigarette Dependence Scale (*abbreviated 5-item scale*), *FTND* Fagerström Test for Nicotine Dependence.

### Paired *t*-tests

Pairwise comparisons for craving and pleasantness ratings are shown in Table 2. The control condition did not lead to any change, neither in craving (*t*(32) = 0.64; *p* = 0.525) nor in picture evaluation total score (*t*(32) = 0.28; *p* = 0.781). For picture 2 (an open, full box of cigarettes), a slight but nonsignificant enhancement of craving was noted (+3.33%; *t*(32) = 1.82; *p* = 0.078).

**Table 2:** Pre-post comparisons across conditions for craving and pleasantness ratings.

	Craving			Pleasantness (pictures)		
	Pre	Post	Statistics	Pre	Post	Statistics
Control	42.42 (5.42)	43.64 (5.74)	*t*(32) = 0.64, *p* = 0.525	51.01 (5.19)	50.61 (5.25)	*t*(32) = 0.28, *p* = 0.781
Thought suppression	31.14 (4.88)	29.43 (4.69)	*t*(34) = 0.97, *p* = 0.338	40.67 (4.53)	35.90 (4.12)	*t*(34) = 3.13, *p* = 0.004
Zooming	27.86 (6.01)	26.07 (5.92)	*t*(27) = 1.72, *p* = 0.096	38.81 (4.61)	37.02 (4.65)	*t*(27) = 0.66, *p* = 0.515
Retraining with movement	27.33 (5.23)	25.33 (5.31)	*t*(29) = 0.64, *p* = 0.527	39.11 (5.44)	33.78 (4.94)	*t*(29) = 1.27, *p* = 0.214
Retraining without movement	34.24 (5.49)	30.00 (5.50)	*t*(29) = 2.30, *p* = 0.028	47.37 (4.28)	44.34 (4.72)	*t*(32) = 1.50, *p* = 0.143
Counter-movement	30.36 (5.65)	28. 57 (5.60)	*t*(27) = 1.15, *p* = 0.259	46.19 (5.03)	44.40 (5.49)	*t*(27) = 0.99, *p* = 0.331

Means standard errors.

Zooming out reduced craving at a statistical trend level (–1.72%; *t*(27) = 1.79; *p* = 0.096) but did not change the pleasantness ratings for any of the pictures or the total score (*p* > 0.30).

A somewhat opposite pattern was found for thought suppression. Here, no effect emerged for craving (–1.71%; *t*(34) = 0.97; *p* = 0.338), but the pleasantness ratings for the smoking-related pictures were significantly reduced (–4.76%; *t*(34) = 3.13; *p* = 0.004). Item-wise analyses of the pictures showed a significant reduction for item 1 (i.e., lit cigarette; –4.86; *t*(34) = 2.19; *p* = 0.036) and item 2 (i.e., open, full box of cigarettes; –4.86%; *t*(34) = 2.22; *p* = 0.033) as well as a trend for picture 3 (i.e., social smoking scene –4.57%; *t*(34) = 1.82; *p* = 0.077).

In the non-motor retraining condition, craving was significantly reduced (–4.24%; *t*(32) = 2.30; *p* = 0.028). The effect of picture appraisal failed to reach significance for the total score (–3.03%; *t*(32) = 1.50; *p* = 0.143). A reduction at a statistical trend level was shown for picture 1 (–6.36%; *t*(32) = 1.99; *p* = 0.055).

In the motor retraining condition, all parameters were reduced numerically (2–6%) but not statistically (*t*(29) <1.5; *p* > 0.15). For exploratory purposes, the retraining conditions were combined as they share some features; reductions were found for craving at a statistical trend (–3.17%; *t*(63) = 1.78; *p* = 0.077) and for a reduction in the pleasantness ratings for the pictures (–4.13%; *t*(63) = 1.84; *p* = 0.071).

The counter-movement condition led to no change in overall picture appraisal and craving (*t*(27) <1.2; *p* > 0.25). Post hoc analyses showed a reduction in the pleasantness rating for the social smoking scene (–5%, *t*(27) = 2.32, *p* = 0.028).

Comparisons among conditions showed a greater reduction in craving (–5.45%; *p* = 0.046) in the non-motor retraining condition relative to the control condition. For picture 1 (a hand holding a lit cigarette), reduction in craving was larger for the motor (–8.12%; *p* = 0.043) and non-motor retraining condition (–8.48%; *p* = 0.030) relative to controls. A combination of the retraining conditions showed a trend for less craving relative to the control condition (–4.39%; *p* = 0.066) and a significant reduction in pleasantness ratings for picture 1 relative to the control condition (–8.31%; *p* = 0.015) and the counter-movement (–7.26%; *p* = 0.044).

We then split the sample into those who had versus those who had not performed the imaginal retraining exercises at some point in the period between baseline and follow-up. Individuals in the joint retraining group who had performed the exercises generally showed a stronger reduction in pleasantness ratings of the pictures (–11.59%) than the other group at trend level (–0.40%; *t*(22.78) = 1.87, *p* = 0.074). For thought suppression, this was significant (–9.23% vs. 2.12%; *t*(33) = 2.41, *p* = 0.074). Only 2.7% of the individuals reported noting a change because they assumed that they were expected to note a change, and this was not differently distributed across conditions (*p* = 0.164).

## Discussion

The study provides preliminary evidence that a single therapeutic “dose” of a non-motor variant of imaginal retraining (i.e., putting distance in the mind’s eye from a craved object) can reduce immediate craving and the positive appraisal of addiction-related pictures. To our surprise, this effect was somewhat stronger for the condition where this inner distancing was not augmented by a corresponding movement. We will turn to the question of why this might be the case later. The zooming out of the imagined cigarette was accompanied by a reduction in craving at a small effect size (trend level), while, as expected, the control condition did not lead to a reduction. Thought suppression had no anti-craving effect but did lead to a less favorable appraisal of smoking-related pictures. When the two core retraining conditions (mental distancing with and without movement) were combined, the comparison to the neutral condition achieved a trend for lower pleasantness ratings of smoking-related pictures and craving. In addition, very few participants affirmed that they had reported a change because they felt that this would be expected of them (no differences across groups).

While the results hold promise, the study has a number of limitations rendering its results preliminary. First, the effect sizes were small and we can only speculate whether the effect would have been stronger if the exercises were repeated—prior findings indeed speak for a dose-effect relationship^21,23^. The effects would not have survived a strict adjustment for multiple comparisons. However, detecting an effect after a single dose is encouraging and suggests that the technique may be effective as an acute anti-craving self-help technique. Second, we found that thought suppression had a small effect on the reduction of craving. This was unexpected. Whether a rebound effect occurs after some time has not yet been studied^32^. Third, the study was part of an intervention trial, and a subgroup of the participants had used the technique before. There is some evidence that this affected the outcome. Those who had used the technique before showed somewhat better results than those who were naive to the intervention. We think that performing the technique or components of it requires some skill and a certain level of acquaintance with the underlying rationale (see below). And fourth, we only tested a subset of components and did not consider others. For example, we did not examine the effect of mood induction. Also, the imaginal component was different from imaginal retraining where an idiosyncratic image is contemplated, not a particular image presented on a screen.

For future studies, we suggest modifying and extending the current design to examine whether the repeated rehearsal of the therapeutic components augments the effects. We also suggest combining components; we expect that some sequences may in fact weaken the outcome because the techniques might be too complex (and hence perhaps poorly executed) or because the impact of an effective component is diluted by an ineffective one. This brings us back to the counterintuitive finding that the non-motor retraining component emerged as slightly more effective than the motor version. We suspect that the former technique may lead to a higher focus on the essential element (namely, pushing away an object in one’s thought), whereas the latter technique introduces distraction due to the need for additional perceptual and motor attention. Of note, motor imagery has long been conceptualized as an internal rehearsal of simple as well as complex motor movements without overt physical action^42,43^; in line with this, there is substantial overlap between the neural correlates of motor imagery with motor execution^44,45^. Thus, both techniques (actual movement and imagined movement) likely map onto similar brain areas. Also, the instructions for the motor technique are much longer than for the non-motor (see the “Methods” section: 78 words compared to 59 words in the imaginal retraining conditions and 18 words in the control condition) and thus perhaps more complicated and prone to errors of interpretation (one reviewer also raised the possibility that the actual movement may seem unnatural, thus weakening the effect). While our findings are thus not fully congruent with a strong formulation of the embodiment account that assumes a pivotal role of the motor system in approach-avoidance behavior, we should delay a decision on this question until replication and also look at the accuracy of the implementation of the training components and their rehearsal. Results suggest that some prior practice of the technique may indeed foster effectiveness. In the current (full) protocol of imaginal retraining, we suggest that participants use smartphone timers to remind them to perform the technique as there is increasing support that frequent practice augments the outcome^21,23^. Future studies may also adopt a more balanced factorial design. We refrained from doing so mainly for ethical reasons such as the possibility that zooming in on an imagined cigarette might increase craving. Future studies should examine active control conditions, for example defusing or other techniques from acceptance and commitment therapy. In addition, studies should test for the effect of mood induction, another element of the imaginal retraining protocol. Trials should also investigate moderators such as expectancy effects and subjective credibility of the intervention. Apart from craving, other outcome measures such as smoking behavior, approach bias, and sustainability of the effect should be tested.

In conclusion, the present study lends first support to the notion that the *avoidance component* of imaginal retraining is immediately effective even after a single “dose.” Although the effect was not strong (craving reduction of −4.24%), it might be sufficient for some individuals to override the momentary urge to smoke cigarettes and might be strengthened upon repetition of the technique.

## Supplementary information

Appendix
