# Supplementary material for: A dismantling study on imaginal retraining in smokers
Source: Transl Psychiatry. 2021 Feb 2;11:92. doi: 10.1038/s41398-020-01191-9 (PMC7854587; doi:10.1038/s41398-020-01191-9)
Supplement: Supplementary file 1 — Appendix [file 41398_2020_1191_MOESM1_ESM.docx]

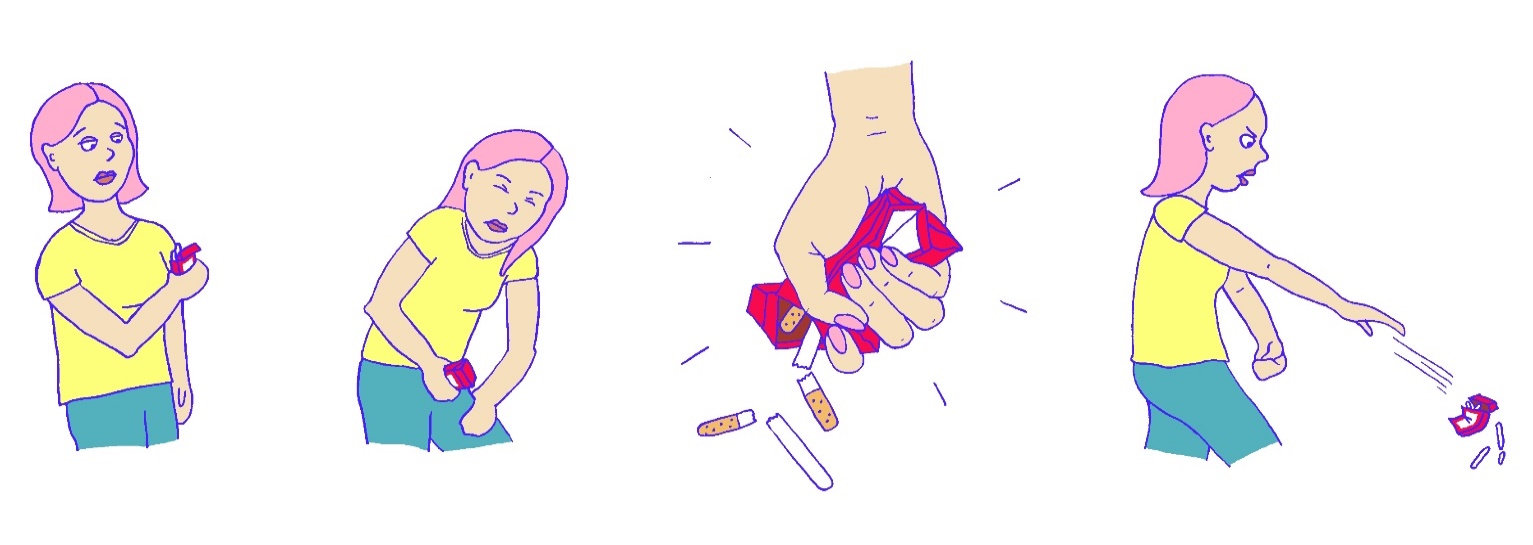
Appendix


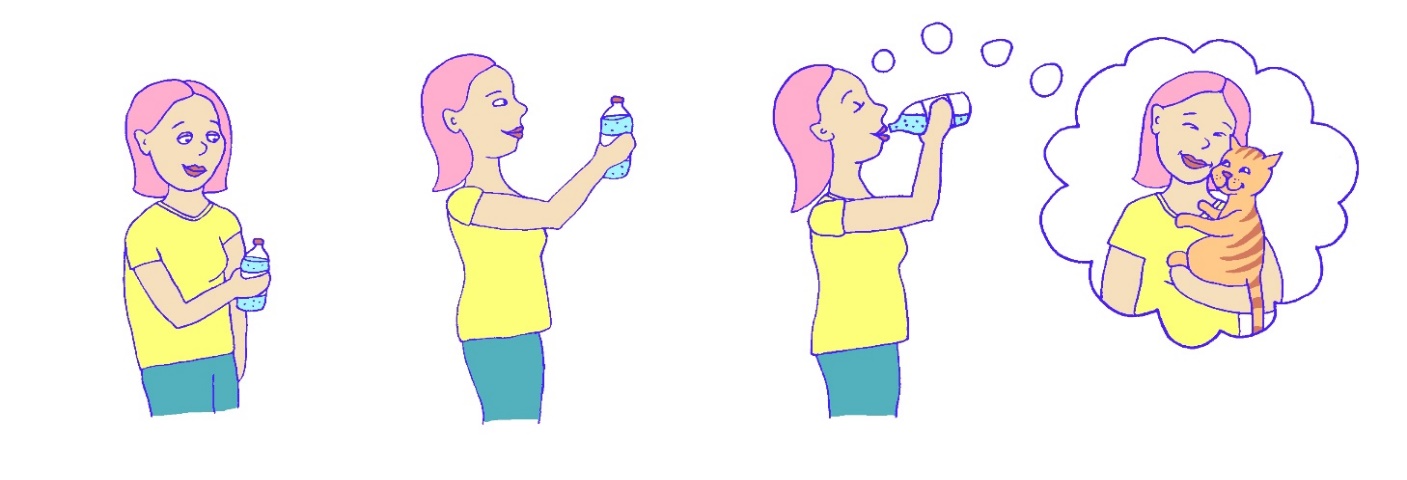


Appendix:

The two steps of imaginal retraining:

Aversion sequence (upper panel): The individual imagines grabbing a cigarette pack of his or her favorite brand, bending forward and contemplating negative thoughts (negative mood induction; the latter component was not tested in this study), and then throwing the imagined pack down and away (actual and imagined behavior).

Approach sequence (lower panel): The individual imagines grabbing a nonalcoholic beverage or healthy food such as a glass of water or an apple, stands upright, lifts the imagined drink high, and, if possible, couples this with other positive feelings (e.g., stroking a pet; actual behavior with imagined objects; the latter component was not tested in this study).

Pictures to be rated (a lit cigarette; an open, full box of cigarettes; and a social smoking scene)

| 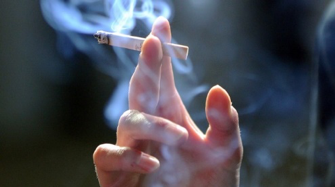 | 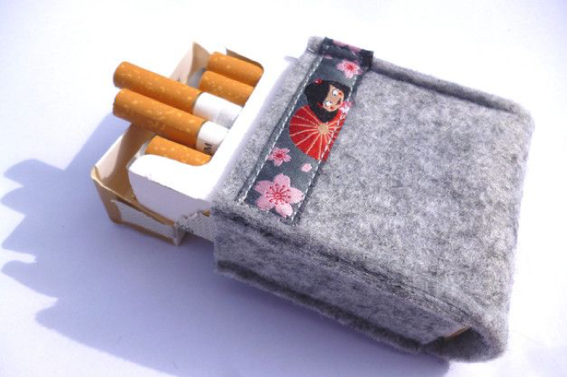 | 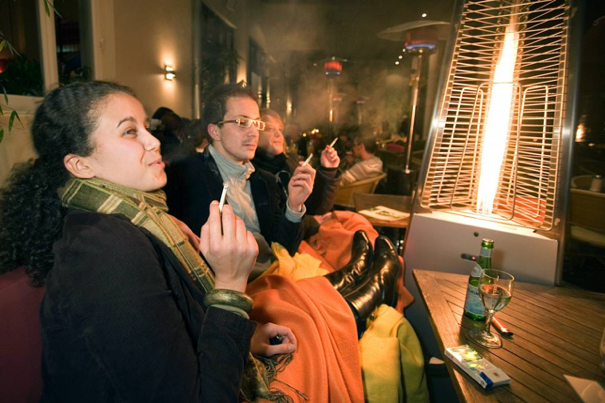 |
| --- | --- | --- |

Experimental conditions:

| 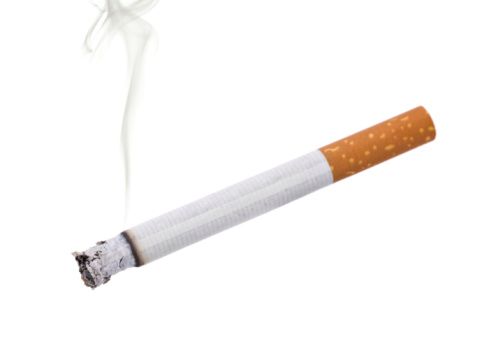  Image shown for conditions 1–5 (see Experimental conditions section) | 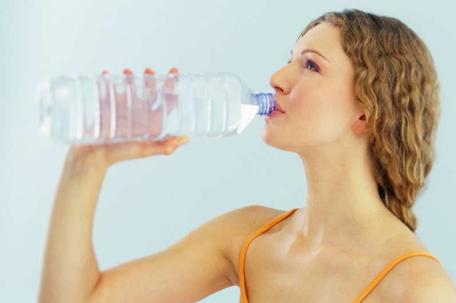  Image shown for condition 6 (see Experimental conditions section) |
| --- | --- |
